# Consequences of early life stress on genomic landscape of H3K4me3 in prefrontal cortex of adult mice

**DOI:** 10.1186/s12864-018-4479-2

**Published:** 2018-02-09

**Authors:** Nikita I. Ershov, Natalya P. Bondar, Arina A. Lepeshko, Vasiliy V. Reshetnikov, Julia A. Ryabushkina, Tatiana I. Merkulova

**Affiliations:** 1 Laboratory of Gene Expression Regulation, Institute of Cytology and Genetics, Siberian Branch of Russian Academy of Sciences, 10 Prospect Lavrentyeva, 630090 Novosibirsk, Russia; 2 Novosibirsk National Research State University, 2 Pirogov Street, 630090 Novosibirsk, Russia

**Keywords:** ChIP-seq, Early life stress, Maternal separation, Handling, H3K4me3, Prefrontal cortex, Mice

## Abstract

**Background:**

Maternal separation models in rodents are widely used to establish molecular mechanisms underlying prolonged effects of early life adversity on neurobiological and behavioral outcomes in adulthood. However, global epigenetic signatures following early life stress in these models remain unclear.

**Results:**

In this study, we carried out a ChIP-seq analysis of H3K4 trimethylation profile in the prefrontal cortex of adult male mice with a history of early life stress. Two types of stress were used: prolonged separation of pups from their mothers (for 3 h once a day, maternal separation, MS) and brief separation (for 15 min once a day, handling, HD). Adult offspring in the MS group demonstrated reduced locomotor activity in the open field test accompanied by reduced exploratory activity, while the HD group showed decreased anxiety-like behavior only. In a group of maternal separation, we have found a small number (45) of slightly up-regulated peaks, corresponding to promoters of 70 genes, while no changes were observed in a group of handling. Among the genes whose promoters have differential enrichment of H3K4me3, the most relevant ones participate in gene expression regulation, modulation of chromatin structure and mRNA processing. For two genes, *Ddias* and *Pip4k2a*, increased H3K4me3 levels were associated with the increased mRNA expression in MS group.

**Conclusion:**

The distribution of H3K4me3 in prefrontal cortex showed relatively low variability across all individuals, and only some subtle changes were revealed in mice with a history of early life stress. It is possible that the observed long-lasting behavioral alterations induced by maternal separation are mediated by other epigenetic mechanisms, or other brain structures are responsible for these effects.

**Electronic supplementary material:**

The online version of this article (10.1186/s12864-018-4479-2) contains supplementary material, which is available to authorized users.

## Background

A growing body of findings underlines the critical role of the early environment in the development of the nervous system and for the behavioral phenotype in later life [1, 2]. The adverse early life events including stress or maltreatment are known to have long-lasting effects on brain function, cognitive and emotional development and can influence the risk to develop stress-related psychopathology in adulthood [2–4]. Clinical studies have revealed a strong link between childhood maltreatment and the development the risk of psychiatric disorders and health risk behaviors including smoking, overeating, and substance abuse [4–7].

However, human studies have some limits to reveal the molecular mechanisms underlying long lasting effects of early life adversity on cognitive functions and behavior. So, various animal models have been developed to investigate the molecular consequences of early life stressful events. Among them, maternal separation models in rodents are the most commonly used and established ones [8–10]. These models have contributed significantly to our knowledge of a link between early life exposures and neurobiological and behavioral outcomes in adulthood. In particular, studies on rats demonstrated that a brief maternal separation (15 min per day; handling, HD) followed by increased maternal care positively affects the development of the offspring, leading to reduced anxiety, increased exploration and communicative behavior. At the same time, prolonged maternal separation (3 h per day; MS) causes significant amounts of stress resulting in negative long-lasting changes of emotion-related behavior, stress reactivity and cognitive functions [11–14]. Data on the effects of MS and HD on behavioral phenotypes, long-term memory and learning in mice are sparse and often inconsistent [15–17]. However, most works agree in that in mice, as in rats, MS leads to anxiety-related behaviors and increased stress reactivity [16–19].

Epigenetic processes such as DNA methylation and post-translational histone modifications are known to play key roles in memorizing of environmental influences [20, 21]. In this way, epigenetic modifications could potentially mediate embedding early social exposure in the genome and provide the long-lasting nature of induced changes in neurobiology and behavior [4, 22, 23]. Therefore, a number of studies have assessed the epigenetic alterations following early postnatal stress, including maternal separation. However, until recently the focus has been on individual genes, mainly involved in the regulation of hypothalamic-pituitary-adrenal (HPA) axis. For example, in rats subjected to maternal separation significant reduction in histone H3 acetylation at glucocorticoid receptor (GR) promoter I_7_ and significant decreases in total and exon I_7_ GR mRNA levels were observed in the hippocampus during adulthood [24]. In contrast, increased H3 acetylation and hypomethylation of the *Crh* promoter region underlying the upregulation of *Crh* was demonstrated in the hippocampal CA1 region of the adult rat with postnatal MS [25]. Similarly, Chen and colleagues reported hypomethylation of the *Crh* promoter in the PVN of maternally deprived adult rats that correlates with enhanced *Crh* transcriptional response to stress in adulthood [26]. Generally, these findings confirm the idea that early life stress induces epigenetic signatures that can lead to changes in gene expression and behaviors.

Since histone modifications can mediate physiological consequences in response to environmental pressure in early life [27, 28], we undertook a genome-wide investigation of the histone H3K4 trimethylation, a key epigenetic mark associated with transcriptional activation, in the prefrontal cortex (PFC) of adult maternally separated (MS or HD) male mice compared to control ones. Given the fact that PFC is responsible for complex cognitive functions, regulation of emotion, and adaptation to stress, we expected to reveal some alterations in promoter-associated H3K4me3 peaks in this brain region reflecting the long-lasting consequences of stress in early life.

## Methods

### Animals

C57BL/6 mice were housed in the Center for Genetic Resources of Laboratory Animals (RFMEFI61914X0005 and RFMEFI62114Х0010), Institute of Cytology and Genetics, the Siberian Branch of the Russian Academy of Sciences, Novosibirsk, Russia. The animals were housed under the standard conditions (12/12 h light/dark regime, switch-on at 7.30 a.m.; food and water available ad libitum). Bottom of the cage was covered with litter. Pregnant females were provided with paper towels for nest building.

### Maternal separation procedure

**Fig. 1:**
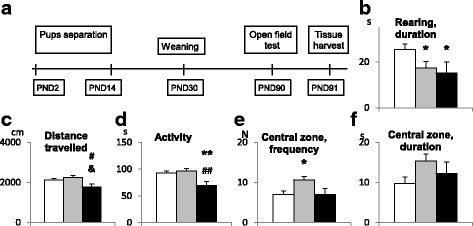
**a** Time line of experiment; **b-f** Effect of early life stress on behavior of adult mice in open field test. White box - control, gray box - HD group, black box - MS group. & - *p* < 0.1 (tendency), * - *p* < 0.05, ** - *p* < 0.01 to compare with controls; # - p < 0.05, ## - p < 0.01 to compare HD group

Nine adult females and nine adult males were mated. Pregnant females were individually housed with paper nesting material during their third week of gestation. Only litters containing 3–6 pups were used for experiments. Nine litters were randomly assigned between three experimental groups: 3 to MS, 3 to HD and 3 to the control group. Offspring were separated from dams on PND 2 through PND 14 (the day of birth was PND 0). Each dam was removed from her home cage and placed into a clean cage. Pups were then removed from its home cage and placed into a small box filled with bedding. After that the dam was placed back into the home cage. The HD pups were separated from their dams for 15 min once a day, while the MS pups, for 180 min once a day. The temperature in the MS cages with pups was kept at 31 ± 2 °C using infrared heat lamps to prevent thermoregulatory distress. No heat lamps were used on HD pups. The control pups were not separated from their dams. After weaning on PND 30, the offspring were housed in groups of 2 to 4 animals of the same sex under standard housing conditions. All offspring (27 males: 11 control, 10 HD, 6 MS) were used in behavioral test and real-time PCR. For ChIP-seq studies 4 animals from each group were used. Time line of experiment is shown on Fig. 1a. Tissue samples were collected for all experimental groups simultaneously, in the morning from 10 till 12 a.m.

### Open field test

Open field test was conducted at PND95 – PND110. Animals were individually housed for 3 days before the test. The test was started at 1 p.m., in the middle of light phase. Open field (OF) consisted of a square arena (80 × 80 cm) with white floor and 25-cm-high walls. The arena was lit by bright light and divided into central zone (40 × 40 cm) and peripheral zone (20 cm from the walls). Each mouse was placed individually in the center of the OF arena, and the following behavioral parameters were recorded during 5-min test period: the total distance travelled, latency of the first exit from the central zone, frequency of central zone visits, time spent in central and peripheral zones; the number of rearing and self-grooming episodes; total activity (Ethovision threshold for activity: 0.2%). The OF arena was thoroughly cleaned between tests with different animals. OF behavior was monitored and scored using an automated tracking system (Noldus Ethovision 10.0, Noldus Information Technology, The Netherlands).

### Chromatin immunoprecipitation

Four mice from each group (control, HD and MS) were chosen randomly for chromatin immunoprecipitation with antibodies to H3K4me3 (ab8580, Abcam). Animals were euthanized 24 h after the OF test. Prefrontal cortex were dissected and stored at − 70 °C until further processed. Chromatin immunoprecipitation were carried according to the method [29] with some modifications. To account for possible batch effects, experimental groups were randomized during the preparation of immunoprecipitation. For DNA fragmentation microccocal nuclease (MNase), that selectively spares nucleosome-bound genomic DNA, was used. Each sample (about 20 mg of murine prefrontal cortex) was homogenized in douncing buffer (10 mМ Tris-HCl pH 7.5, 4 mM MgCl_2_, 1 mM CaCl_2_) containing Halt Protease Inhibitor Cocktail (Thermo Scientific, USA) and then incubated with 1 μl of MNase (2000 gels units, NEB, USA) for 6 min at 37 °C. Reaction was stopped by adding EDTA to 10 mM. 4-fold volume excess of hypotonic lysis buffer (0.2 mM EDTA, 1.5 mМ DTT, Halt Protease Inhibitor Cocktail) was added to each sample and samples were incubated on ice for 60 min, with a brief vortexing at 10 min intervals. Debris is then removed by centrifugation at 3000×*g*. To reduce non-specific binding and background impurity (pre-clearing of samples) G-protein magnetic beads (NEB, USA) and 10X IP buffer (200 mM Tris HCl (pH 8.0), 20 mM EDTA, 1,5 M NaCl) were added to samples and incubated at 4 °C for 2 h with rotation. Then magnetic beads with non-specific binding fragments were collected using the magnetic stand and discarded. 10% of pre-cleared sample was saved as input DNA (Inp) for subsequent ChIP enrichment analysis. At the same time H3K4me3 antibody (3 μl) were incubate with G-protein magnetic beads with rotation at 4 °C for 2 h. Supernatant were discarded from antibody-coated beads, beads were combined with pre-cleared chromatin and incubated overnight at 4 °C with rotation. After that beads together with the captured immune complexes were washed twice by 1 ml Low Salt (0.1% SDS, 1% Тriton Х-100, 2 mМ EDTA, 20 mM Тris-HCl (рН 8.0), 150 mM NaCl), High Salt (0.1% SDS, 1% Тriton Х-100, 2 mМ EDTA, 20 mМ Тris-HCl (рН 8.0), 500 mМ NaCl), LiCl (1% IGEPAL-СА 630, 1% deoxycholic acid, 1 mМ EDTA (рН 8.0), 10 mМ Тris-HCl (рН 8.0), 0.25 М LiCl) and TE (10 mМ Тris-HCl (рН 8.0), 1 mМ EDTA) buffers. Captured IP-complexes were eluted by incubation for 1 h at 65 °C in 50 μl of freshly prepared elution buffer (100 mМ NaHCO3, 1% SDS). Supernatant was transferred in a new tube. After that, beads were washed by 50 μl of elution buffer and supernatant was collected in the same tube. Samples and input DNA were proteinase K and RNaseA treated, and then purified using phenol chloroform extraction, and DNA finally were eluted in 30 μl of 4 mM Tris-HCl (pH 8.0).

Quantitative real time PCR was carried out for each sample and inputs with positive and negative control primers for ChIP enrichment analysis. The promoter region of *Rpl30*, located within the active chromatin and enriched with H3K4me3 according to the UCSC Genome Browser data was selected as a positive control. The promoter region of *Oosp3*, which is expressed only in oocytes and is not enriched with H3K4me3 in prefrontal cortex of mice (UCSC Genome Browser data), was used as a negative control. The following primer pairs were designed: *Rpl30* forward – 5-ACTTTGCACAGGGACCACAA, reverse – 5-TTACCCGTCAGCCACTTCAC; *Oosp3* forward – 5-ACAGCATTGTGTCAGCATCCCTAAA, reverse – 5-GCCTGAATATGCTTGTCTAGGTGGC. Fold enrichment (FE) was estimated for each sample: FE = 2^(Ct Inp – Ct IP) pos^/2^(Ct Inp – Ct IP) neg^. Only samples with FE > 25 were used for library preparation.

### Library preparation

Libraries were prepared according to standard New England Biolabs protocols (USA). The DNA was end-repaired using T4 DNA polymerase and Klenow DNA polymerase. After that, an A base was added to the 3′ end of the blunt phosphorylated DNA fragments, and an Illumina adaptor with a single T overhang at its 3′ end was then ligated to the end of the DNA fragment. Then USER™ enzyme was added for cutting closed adaptors. Size selection of DNA fragments was performed by means of Agencourt AMPure XP Beads (Beckman Coulter) and then PCR enrichment of the adapter-ligated library was conducted (eight cycles of PCR). The size and quantity of the library were verified on the Agilent Bioanalyser. Preliminary low-depth sequencing using the MiSeq Illumina was essential for correct quantity determination. Subsequent paired-end (2 × 100) sequencing was performed using the Illumina HiSeq2500 (Ltd Genotek, Moscow, Russia).

### ChIP-seq data analysis

On average, ~ 10 million paired-end reads (8.8–10.4 million) were obtained for each sample. Paired-end sequencing data were preprocessed by trimmomatic [30] adapter removal tool and mapped to GRCm38/mm10 mouse reference genome using bowtie2 aligner [31]. The quality metrics of ChIP-seq libraries (Additional file 1: Table S1) were assessed by phantompeakqualtools software (https://www.encodeproject.org/software/phantompeakqualtools/). MACS2 [32] algorithm with nucleosome-optimized parameters (−-shift 37 --extsize 73) was applied to call both broad and narrow peaks from the pooled data. Public sequencing data on MNase-treated input samples of mouse PFC neurons (NCBI SRA accessions: SRR5032624, SRR1647895) were served as background in peak calling procedure. GENCODE comprehensive gene annotation release M13 (GRCm38.p5, https://www.gencodegenes.org) was used to link the predicted peaks to the nearest TSS of genes. An analysis of individual variability in ChIP-seq data was performed in R programming environment (https://cran.r-project.org) on a subset of broad H3K4me3 peaks located in promoter regions (±1Kb around TSS of annotated genes) and required to have a peak-calling q-value ≤0.01.

For differential enrichment (DE) analysis, DESeq2 [33] and csaw [34] R packages were used. In DESeq2 workflow, reads with mapping quality (MAPQ) of at least 10 were counted at the predicted peak regions. Peaks overlapping the DAC blacklisted regions (ENCODE accession ENCFF547MET) were excluded from the subsequent analysis. Normalization, modeling, and statistical testing were done with default parameters. At most 10% FDR (assessed by Benjamini–Hochberg procedure) was allowed in the resulting list of DE peaks. Two additional filtering criteria, the location in promoter regions of known genes (±1Kbp around TSS) and the absence of substantial input bias in the corresponding locus, were applied to produce the final list of DE peaks.

Similarly, in csaw workflow, reads with MAPQ> 10 were counted in 150-bp genomic windows excluding the ones from DAC blacklisted regions. Count data were filtered by 5-fold change over global enrichment and normalized on efficiency bias. Subsequent statistical testing with controlling 10% FDR did not reveal any DE windows in the data.

### Pathway analysis

Gene set enrichment and functional network analyses were performed with the use of GSEA [35], DAVID [36], MSigDB [37], and STRING databases [38].

### RNA extraction

Frozen tissue samples of prefrontal cortex were treated with TRIzol Reagent (Ambion, USA) following the manufacturer’s instructions and the total isolated RNA was purified by Agencourt RNAClean XP Beads (Beckman Coulter). In each sample RNA quality and yield was confirmed by NanoDrop 2000. One microgram of the total RNA was taken for cDNA synthesis using a reverse transcription kit (Syntol, Russia), with a random hexanucleotide mixture as primers. All procedures were carried out according to manufacturer’s instructions.

### Real time PCR

A thermocycler CFX96 (Bio-Rad, USA) was applied for quantitative real-time PCR using TaqMan probes. Primers and TaqMan probes (Syntol, Russia) were designed (Additional file 2: Table S2) using the Primer BLAST (NCBI). The reaction mixture consisted of 0.25 mM dNTP, 2 μl 10Х PCR AS buffer-B (15 mM MgCl2, 650 mM Tris-HCl (pH 8.9), 160 mM (NH4)2SO4, 0.5% Tween 20), 10 pmol of each forward and reverse primers, 10 pmol TaqMan probes, 4 μg cDNA sample, 0.3 U SynTaq polymerase (Syntol, Russia), and ddH2O to a final volume of 20 μl. The PCR program consisted of 39 cycles of 95 °C for 30 s, and 60 °C for 30 s. All qPCR reactions were performed in duplicates. The qPCR data were analyzed using the ΔΔCt method and normalized to the housekeeping genes hypoxanthine guanine phosphoribosyl transferase (*Hprt1*) and ribosomal protein 16S (*Rpl16S*). The stability of the two housekeeping genes between and within groups was verified using the Bio-Rad CFX Manager software (Bio-Rad, USA) with the gene stability value M, set at less than 0.5 and coefficients of variation CV, at less than 0.25.

## Results

### Open field behavior

In OF test, maternal separation had the greatest effect on the locomotor activity of animals (Fig. 1b–f). One way ANOVA revealed the influence of stress on distance travelled [F(2,24) = 3.6; *p* = 0.043] and total activity [F(2,24) = 5.2; *p* = 0.013]. MS group demonstrated significantly less distance travelled compared with HD group (*p* = 0.013) and control (a trend, *p* = 0.074). Decreased locomotor activity is confirmed by a lower level of total activity in MS group compared to control (*p* = 0.0096) and HD (*p* = 0.004) groups. The decreased locomotor activity accompanied by reduced exploratory activity [F(2,24) = 4.9; *p* = 0.017] – the duration of vertical movements (rearings) was lower in MS group (*p =* 0.023) than in the controls. HD mice showed normal locomotor activity but increased frequency of entries into the central zone of the open field (*p* = 0.017) compared with control, which indicates a decreased anxiety level in HD group [F(2,24) = 3.7; *p* = 0.039]. HD group also showed a reduced exploratory activity estimated by decreased duration of rearings (*p* = 0.014).

### ChIP-seq QC

To assess whether the adults with a history of early life stress possess any long-term changes in H3K4me3 profile of PFC, we performed the native MNase-treated ChIP-seq analysis targeting the studied histone mark in mice subjected to brief and prolonged maternal separation and control animals. The approach produces histone profiles with a much better signal-to-noise-ratio compared to protocols utilizing formaldehyde fixation [29]. All the libraries except one (discarded) were sequenced at sufficient depth and demonstrated high enrichment values as well as passed all the other quality estimators recommended by ENCODE Consortium [39] (Additional file 1: Table S1).

**Fig. 2:**
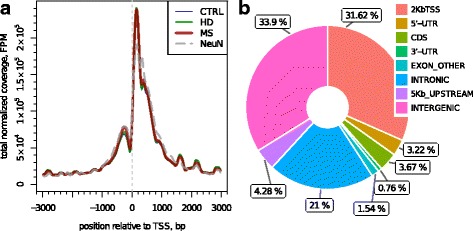
Genomic distribution of H3K4me3 peaks detected in PFC. **a** Enrichment of H3K4me3 around TSSs. The data are normalized on total fragments mapped to TSS regions and pooled within experimental groups (CTRL, HD, MS); available data on H3K4me3 in NeuN-positive PFC neurons (SRR5032626) is provided for comparison purposes. **b** Distribution of peaks across different types of genomic regions

The called H3K4me3 peaks were located predominantly in the promoter regions of genes and formed the typical shape around the TSS (Fig. 2), fully resembling the genomic distribution described earlier [40]. We utilized the publicly available input data on mouse PFC neurons for peak-calling. While the use of such input control may affect the FDR of peak detection, it should not have any pronounced effect on a comparative analysis of differential enrichment of detected peaks. Given the data successfully resembled all known features of H3K4me3 mark, we considered the use of third-party input control acceptable for comparative study.

### Individual variability

**Fig. 3:**
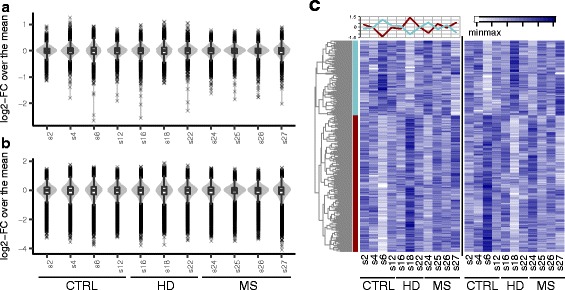
Variation of H3K4me3 profiles across the samples. **a, b** Violin plots of sample-wise distribution of fold-changes over the mean peak enrichment of all samples. The data received on broad (**a**) and nucleosome-sized (**b**) peaks are presented. **c** A heatmap of enrichment values for a subset of 718 co-regulated peaks showing highest variability across the samples (left panel). For each peak on the left panel, the corresponding highest negatively correlated peak is provided on the right panel, allowing for duplicates. Two oppositely varying clusters of peaks are observed. Dynamics of the mean enrichment in the two clusters is shown at the top of the left panel

To explore the variability of obtained H3K4me3 profiles across individuals, we extracted a set of 12,112 highly reliable broad peaks (MACS q-value <= 0.01) located in promoter regions of annotated genes. To get more detailed information about the sources of variation, two different resolutions were used to collect the normalized enrichment values, i.e. composite broad peaks and their constituent nucleosome-sized peaks (68,177). The broad peaks showed a remarkably low variability across all the samples, with the vast majority of sites not reaching a two-fold difference from the mean (Fig. 3a). However, the nucleosome-sized peaks were clearly more variable (Fig. 3b), indicating higher fluctuations in the shape of the corresponding broad peaks than in their cumulative enrichment values. It should be noted that the observed prevalence of down-regulated outliers is associated with the bias of fold-change metric when the values are near the lower bound of ChIP-seq detection range.

A correlation analysis performed on 1000 most variable broad peaks showed that most of them correlated significantly (r > =0.8, *p*-value<=0.01) with each other. Since positive correlation alone may arise from systemic technical bias, we further extracted a subset of negatively correlated peaks, each required also to have a positive correlation within a given subset. Seven hundred eighteen peaks met the requirements and were subsequently split into 2 opposing clusters (Fig. 3c). The latter result assumes that the fluctuations in the most variable peaks are mainly caused by a single factor. Respectively, PCA analysis on a whole set of 12,112 broad peaks showed that the corresponding PC1 factor explains 27% of observed variation (and 53% in a subset of 718 peaks). However, it does not correspond to any of experimentally controlled factors, e.g. stress condition, litter/kinship, or library enrichment.

We then assessed whether the observed covariation of H3K4me3 peaks is functionally meaningful. Functional enrichment analysis using MSigDB and DAVID resources showed high functional relatedness and tight association of corresponding genes within the clusters and a clear distinction between them (Additional file 3: Table S3). In particular, the first cluster is overrepresented by neuron-specific genes related to axon guidance, synapse assembly, and synaptic (including GABAergic and glutamatergic) transmission. The second cluster is enriched with more general nuclear transcription factors and chromatin regulators, including several histone modifiers (*Mll2*, *Nsd1*, *Setd1b*, *Ash1l*, *Ezh2*). Thus, the observed low individual variation appears to be functionally relevant and is partially described by a single factor, albeit not directly related to the factor of stress conditions.

### Chip-seq DE analysis

To analyze the differential enrichment of H3K4me3 profiles we used both peak-based MACS-DESeq2 and window-based csaw workflows; both make use of biological replicates in statistical testing, and the latter does not require peak regions as a prerequisite. Csaw didn’t reveal any statistically significant changes in both experimental groups versus controls. The DESeq2-based approach also failed to detect any in a group of handling, but detected a small subset of modestly altered broad peaks in a MS group (Additional file 4: Table S4). 45 regions of H3K4me3 modification were found to be increased, affecting promoters of a total of 70 genes. Thereby, a substantial part of the differentially up-regulated marks (42%) was detected within the bidirectional promoters.

**Fig. 4:**
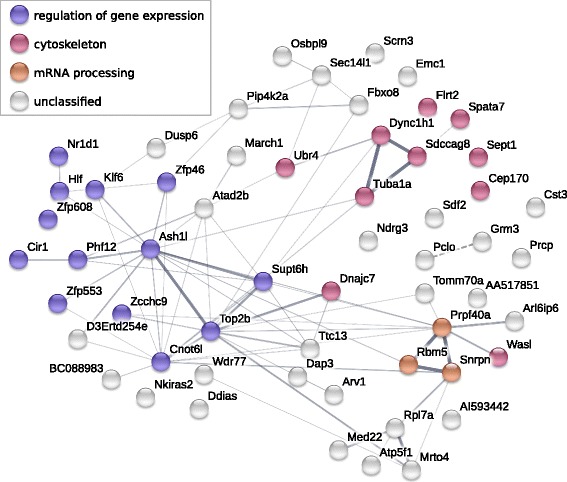
STRING network of interactions between the genes differentially enriched by H3K4me3 in MS group. Color of nodes represents grouping by enriched GO terms

Since the observed fold changes of the differentially enriched regions are quite low, we have assayed whether the corresponding genes share any common functional features, thereby supporting non-randomness of their identification. The STRING network reconstruction and GO enrichment analysis allowed to combine a number of genes into certain, albeit somewhat general, functional groups (Fig. 4). The broadest and most densely connected one was related to the regulation of gene expression (GO:0010468, adjP = 0.0435), including genes associated with modulation of chromatin structure (*Ash1l*, *Supt6*, *Cir1*, *Phf12* etc), transcription factors (*Klf6*, *Hlf*), e.g. zinc finger proteins (*Zfp608*, *Zfp553*, *Zfp46*, *Zcchc9*), etc. This group is coupled with the genes involved in the mRNA processing (GO:0006397, adjP = 0.0456) – splicing (*Prpf40a, Rbm5*) and degradation (*Cnot6*). Another group of genes with increased levels of trimethylated H3K4 in promoters refer to cytoskeleton (GO:0005856, adjP = 0.0130). Among these, it is worth noting the genes responsible for motility of synaptic vesicles (*Dync1h1*) and dendrite spine formation (*Wasl*) in neurons.

To compare the distribution of over-represented pathways between the stressed groups (MS and HD) we performed a Gene Set Enrichment Analysis (GSEA) of pre-ranked gene lists (using ranking by *p*-value and Reactome gene sets of size 5 to 500). HD group was found to be enriched with the same Reactome pathways as MS group, while the latter showed much broader set of over-represented pathways (140 vs 22, Additional file 5: Table S5). This may indicate the similarity of stress response in both studied groups, either more pronounced in MS group. Common categories are connected to RNA processing and transcription regulation, and in a less degree to metabolic processes. Additionally, MS group was enriched by some neuronal and immune pathways as well as pathways of vesicular and membrane transport.

Thus, the results of the enrichment analysis indicate non-randomness of the revealed weak changes in H3K4me3 profiles in MS group of mice. These also suggest that the consequences of early postnatal stress are manifested in epigenetic changes of genes that are one way or another regulate gene expression.

### qPCR results

**Fig. 5:**
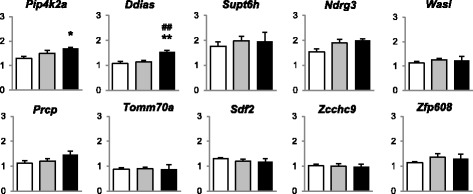
RT-qPCR validation of gene expression. White box - control, gray box - HD group, black box - MS group. mRNA level normalized to *Hprt1* and *16S*. Data represent as mean ± SEM. *p < 0.05, **p < 0.01 to compare with control group, ##p < 0.01 to compare with HD group (LSD post hoc test)

We have randomly selected 10 out of 70 genes showing significant changes of H3K4me3 enrichment in their promoters, to investigate changes of their expression using real time PCR (Fig. 5). Early life stress was found to influence on expression of only two genes - *Ddias* (*DNA damage-induced apoptosis suppressor*) [F(2,24) = 8.01, *p* = 0.003] and *Pip4k2a* (*phosphatidylinositol-5-phosphate 4-kinase, type II, alpha*) [F(2, 24) = 2.11, *p* = 0.094]. MS mice have a higher expression level of *Pip4k2a* (FC = 1.32, *p* = 0.041) and *Ddias* (FC = 1.43, *p* = 0.001) compared to controls and a higher expression level of *Ddias* (FC = 1.34, p = 0.003) compared to HD group. Thus, the expression levels of the majority of genes remained unaltered, while the changes of the rest were as weak as those of H3K4me3 levels.

## Discussion

In the present study we examined a model of different type of stress in the early life period, which have been shown to have different impact on the behavior of adult mice. Indeed, in our study a prolonged maternal separation (MS) resulted in alterations of individual behavior, namely a stable decrease of locomotor and exploratory activities, while brief maternal separation (HD) induced a decreased anxiety in adulthood. Similar opposing effects of brief and prolonged early life stress were previously shown in behavioral studies on rats [41–43]. In mice, the consequences of MS and HD on behavior are much less studied, with some findings being contradictory. Nevertheless, maternal separation of pups from dams generally increases anxiety [18, 19, 44] and in some cases results in depressive-like behavior of adult male mice [45]. Several studies also demonstrated a decreased anxiety in adult male mice experienced brief maternal separation [17, 46]. Our results confirm the presence of adverse long-term effects of prolonged maternal separation in early life on behavioral traits and beneficial behavioral effects of handling in mice.

There are provisional grounds to consider that the long-term behavioral consequences of early life stress are likely maintained by epigenetic mechanisms. Thus, a number of studies show stable changes in CpG methylation in promoter regions of genes encoding glucocorticoid receptor [45, 47–49], BDNF [45, 50, 51] and others [52] caused by early postnatal stress. Moreover, maternal separation alters the expression levels in conjunction with the levels of histone H3 acetylation and/or some repressive modifications (H3K9me2, H3K9me3) in promoter regions of *GR*, *Crh*, *Bdnf*, etc. [24, 51, 53–56].

The choice of H3K4me3 modification in our study was dictated, on the one hand, by the fact that it is relatively long-lasting (compared to histone acetylation or phosphorylation) to mediate the observed durable changes of behavior [27]. On the other hand, it is tightly associated with promoter regions, thus bringing direct link of its levels with transcriptional activity of the corresponding genes [57, 58]. The maintenance of Mll1-regulated H3K4 methylation at particular genes in PFC was shown to be critical for normal cognitive abilities and emotional state [59]. Besides, its level was shown to be altered in rat hippocampus after the chronic stress while remain unchanged after the acute stress [60].

The profiles of H3K4me3 identified in our study showed surprisingly low interindividual variability. While the source of this variation appeared to be systemic and biologically relevant, it didn’t correspond to the experimental conditions of stress. Consequently, we have found only some subtle changes of H3K4me3 levels that exceeded a relaxed significance threshold (10% FDR) in a group of adult mice experienced prolonged early life stress (MS) compared to controls. Brief separation (HD) did not lead to any significant differences in the distribution of this histone mark at all.

Among the genes, whose promoter regions were found to be differentially trimethylated, there are several ones implicated in neuronal function and chromatin regulation. Thus, the level of H3K4 trimethylation was affected in several genes belonging to glutamatergic neurotransmission. *Grm3* encodes the glutamate receptor mGluR3, a major regulator of PFC function and cognition [61], whose disruption leads to working memory defects [62], as well as development of bipolar disorder [63]. Sdf2 protein was found in a complex with another glutamate receptor, mGluR1b, suggesting its involvement in folding and transport of the latter [64]. *Pclo* encodes the neurospecific Piccolo protein highly expressed in cerebral cortex and participating in synaptic vesicle trafficking. Piccolo plays a pivotal role in synaptic plasticity, controlling the extracellular levels of glutamate [65]. Phosphatidylinositol 4,5-bisphosphate (PtdIns(4,5)P2), generated by enzymatic action of *Pip4k2a* gene product, plays an important role in membrane trafficking, regulation of exocytotic fusion of synaptic vesicles (glutamate, dopamine) with the plasma membrane and regulates a duration of signal transduction [66–68]. Among the other affected genes closely related to neuronal functions, there are *Arl6ip6*, *March1* and *Wasl*, that regulate dendritic spine formation and maintenance [69–71], as well as *Zfp608*, *Flrt2* and *Atad2b*, involved in axonogenesis and neuronal cell differentiation and migration in the developing cortex [72–74].

Furthermore, the presence of several genes associated with chromatin remodeling may indicate the involvement of chromatin architecture in mediating the consequences of early life stress. A histone chaperone *Supt6* is implicated in maintenance of chromatin structure [75, 76]. A product of *Ubr4* appears to be a cytoskeletal component in the cytoplasm and a subunit of the chromatin scaffold in the nucleus [77]. There are also genes related to epigenetic inactivation of transcription, encoding histone methyltransferase *Ash1l*, specifically methylating ‘Lys- 36’ of histone H3 (H3K36me) [78], *Cir1* and *Phf12*, involved in the assembly of histone deacetylase complexes [79, 80], as well as a gene associated with activation of transcription, *Wdr77*, encoding a subunit of methyltransferase complex, which modifies specific arginines to dimethylarginines [81].

However, of 10 genes selected for differential expression analysis by qPCR, the expression of only *Pip4k2a* and *Ddias* was correspondingly increased. It should be noted that, while at the genome-wide level there is usually a strong correlation between gene expression and H3K4me3 levels, the same does not necessarily hold true for any particular gene, since the regulation of gene expression usually depends on numerous factors. Given the small amplitude of observed H3K4me3 changes, we may suppose that they are unlikely to lead to phenotypically pronounced changes in expression of corresponding genes, and thus their contribution to behavioral changes is highly questionable. On the contrary, given the high functional divergence of cortical neurons, we may assume that the relevant changes in a particular set of cells are smoothened by the rest cell mass.

The most likely explanation is that all the prominent changes in H3K4me3 landscape induced by early life stress were transient and restored to the normal levels by this age. Supporting findings were obtained on a mouse model of pronounced cognitive deficits in adult offspring with a history of maternal immune activation during prenatal development mediated by a single dose of polyriboinosinic–polyribocytidilic acid (poly IC) [82]. In spite of the lasting working memory deficits, adult poly IC-exposed mice did not show any significant changes in H3K4me3 epigenetic landscape in the mature cerebral cortex. However the authors demonstrated that H3K4me3 profile in a number of genes was sensitive to acute IL-6 activation in primary cultures from fetal forebrain. Similarly, maternal separation in early life was shown to have no significant effect on the pattern of cortical H3K4me3 in adolescent offspring (PND30) just 2 weeks after the stress exposure [83], although its effects on the behavior and expression of genes being observed in adult offspring [52].

## Conclusion

Summing up, the distribution of H3K4me3 in PFC, even if disturbed by maternal separation during the early life period, seems to stabilize up to a nearly normal state in adult mice, showing relatively low variability across all individuals. It is possible that this modification does not directly implicate in the observed long-lasting behavioral alterations induced by early life stress, or other brain structures are responsible for these effects. Though the possibility that the differences in H3K4me3 can only reappear under the secondary acute stress exposure is not ruled out.

## Additional files

Additional file 1: Table S1. Quality metrics of 12 sequenced ChIP-seq libraries (XLSX 11 kb)

Additional file 2: Table S2. Primers and Taqman probes for real time PCR (DOCX 43 kb)

Additional file 3: Table S3. List of DAVID terms and MSigDB gene sets enriched in two identified clusters of genes whose promoters bear oppositely regulated H3K4me3 mark. (XLSX 34 kb)

Additional file 4: Table S4. H3K4me3 peaks differentially enriched in PFC of MS mice. (XLSX 18 kb)

Additional file 5: Table S5. Reactome pathways significantly up- and dowregulated by stress. (XLSX 24 kb)
