# Supplementary material for: Consequences of early life stress on genomic landscape of H3K4me3 in prefrontal cortex of adult mice
Source: BMC Genomics. 2018 Feb 9;19(Suppl 3):93. doi: 10.1186/s12864-018-4479-2 (PMC5836825; doi:10.1186/s12864-018-4479-2)
Supplement: Supplementary file 2 — Primers and Taqman probes for real time PCR (DOCX 43 kb) [file 12864_2018_4479_MOESM2_ESM.docx]

**Table S1. Primers and Taqman probes for real time PCR**

| **Gene** | **Sequence 5'🡪3'** | **Product size, bp** |
| --- | --- | --- |
| *Ddias* | for ACGGAAATTCCTTTTTGCCTCA | 149 |
|  | rev TGGTACTTCCAGTGTCACCA |  |
|  | probe/56ROXN/TTGGGACAAGTAAACCTTTTGGACT/3BHQ_2/ |  |
| *Ndrg3* | for TGGCAGACTGTGGAGGACTG | 147 |
|  | rev GGGTCATGCTGGCAGATGGT |  |
|  | probe /56-ROXN/CCTGGGAAGCTCACCGAGGCCT/3BHQ_2/ |  |
| *Pip4k2a* | for AATGACTTCATCAATGAGGGCCAAA | 138 |
|  | rev GTGAATCCCCACCAGGAGGC |  |
|  | probe /56-ROXN/ATGTCGAGTTCCTTGCACAGT/3BHQ_2/ |  |
| *Prcp* | for ACGAAGGGGACATTGTCTGG | 117 |
|  | rev AGGGAAGGGACTCCCCATAG |  |
|  | probe /56-ROXN/ATACGGGGTTCATGTGGGATG/3BHQ_2/ |  |
| *Sdf2* | for AGCCACAGTGTGTGAAAGGG | 146 |
|  | rev CTTCTTCGCCAAAGGCACTC |  |
|  | probe /56-ROXN/TCACCTCTCTCTGGAAACCAGGAA/3BHQ_2/ |  |
| *Supt6h* | for TCCTCAAGATCGACACTGCTT | 126 |
|  | rev ATTCTAAGGCATCCACTGCCA |  |
|  | probe /56-ROXN/TCAATGTATGAGTCAGTGCTGTCCC/3BHQ_2/ |  |
| *Tomm*  *70a* | for GTGCAAAAGCCCATGAGAAGC | 95 |
|  | rev GCTCTGCTCATTTTGAAACCC |  |
|  | probe /56-ROXN/TAGAAGACGTCACTGCTGTGTGT/3BHQ_2/ |  |
| *Wasl* | for TGTGGGAACAAGAGCTATACAAT | 144 |
|  | rev GGTCTGTAACTGCTTTTCGGAAC |  |
|  | probe /56-ROXN/AGCTACTTGACAAGTATCTCCAGCA/3BHQ_2/ |  |
| *Zcchc9* | for CTCTGTGGCTCCGTGGAACA | 125 |
|  | rev GGCACATCCAGCACGTCTTC |  |
|  | probe /56-ROXN/ACCAACCGTGATGATCCGATCTGAG/3BHQ_2/ |  |
| *Zfp608* | for AAGCAGACAGGTGTAGACCCA | 107 |
|  | rev TCTGGCTTTTGTTGGTCCAG |  |
|  | probe /56-ROXN/CCATGTCCTTGACTCTGGATCTTGT/3BHQ_2/ |  |
| *Hprt* | for CAAACTTTGCTTTCCCTGGT | 102 |
|  | rev TCTGGCCTGTATCCAACACTTC |  |
|  | probe /56-ROXN/TTGCAAGCTTGCTGGTGAAAAGG/3BHQ_2/ |  |
| *Rps16* | for AGATGATCGAGCCGCGC | 96 |
|  | rev GCTACCAGGGCCTTTGAGATGG |  |
|  | probe /56-ROXN/TGCAGTACAAGTTACTGGAGCCTGT/3BHQ_2/ |  |
